# Thermal Release Characteristics of Aeroengine Combustor Outlet Modulated by Gliding Arc Plasma

**DOI:** 10.1002/advs.202508724

**Published:** 2025-09-23

**Authors:** Lei Zhang, Jinlu Yu, Zhankai Kang, Wenhao Su, Guangxia Chen, Yang Yu

**Affiliations:** ^1^ Aviation Engineering School Air Force Engineering University Xi'an 710038 China

**Keywords:** aeroengine combustor, combustion efficiency, hot‐streak area ratio, gliding arc plasma, outlet temperature profile, outlet temperature distribution factor

## Abstract

A gliding arc plasma (GAP) combustion dome is used to construct a diagnostic platform for the swirl combustor of aeroengines. Thermocouple measurements, particle image velocimetry, and spontaneous emission imaging are employed to measure the outlet temperature field, combustor flow field, and combustion field information. The influence of GAP‐assisted combustion on the outlet temperature profile of the combustor is then analyzed. The results demonstrate that gliding arc discharge allows the flame to reside closer to the combustion dome, shifting the combustion reaction zone forward. This combustion zone contains most of the active particles generated by gliding arc discharge, enabling a more complete combustion reaction and better heat release. The forward movement of the combustion reaction zone also provides sufficient space for flames to develop inside the combustor. The thermal and transport effects of gliding arc discharge enhance the aerodynamic effect inside the combustor, improve fuel atomization, broaden the flame development range inside the combustor, and make the combustion reaction more complete. Under various working conditions, the application of GAP‐assisted combustion increases combustion efficiency by 10.3%, the outlet temperature of the combustor rises by 141.6°C, the outlet temperature distribution function decreases by 29.56%, and the hot‐streak area ratio decreases by 72.6%.

## Graphical abstract

Gliding arc plasma‐assisted combustion significantly improves aeroengine combustor. Gliding arc plasma moves flames closer to the fuel source, enabling complete burning. This increases the combustion efficiency by 10.3% and raises outlet temperature by 141.6°C. Temperature distribution becomes more uniform, while hot spots are dramatically reduced by 72.6%. Gliding arc plasma enhances fuel mixing and burning throughout the combustor.

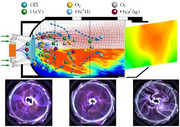

## 1 Introduction

With the rapid development of aviation technology, the thrust‐to‐weight ratio of aeroengine continues to increase. Increasing the outlet temperature of the combustor is the most direct and effective means of achieving a high thrust‐to‐weight ratio.^[^ ^1^, ^2^, ^3^ ^]^ Therefore, high‐temperature‐rise combustors have become an important development direction for future aviation engine combustors.^[^ ^4^ ^]^ The quality of a combustor's outlet temperature field is an important aeroengine performance indicator, directly affecting the performance, lifespan, and reliable operation of turbine components.^[^ ^5^ ^]^ Hence, effectively regulating the outlet temperature profile of the combustor is a key issue in improving the performance of aeroengines.^[^ ^6^ ^]^

The outlet temperature profile of the combustor is influenced by various factors, particularly the geometric structure of the combustor and the coupling of multiphase flow at the combustion dome.^[^ ^7^ ^]^ Hu et al.^[^ ^8^ ^]^ studied the effect of sleeve length on the outlet temperature profile of the combustor, and found that increasing the sleeve length reduced the uniformity of the profile. Wang et al.^[^ ^9^ ^]^ studied the influence of the fuel–air ratio on the outlet temperature profile characteristics of staged combustors, and reported that increasing the fuel–air ratio worsened the uniformity of the combustor outlet temperature field. Holdeman et al. found that the arrangement and shape of the mixing holes^[^ ^10^, ^11^, ^12^ ^]^ can affect the outlet temperature profile of the combustor, and provided relevant empirical formulas. The swirl direction,^[^ ^13^ ^]^ swirl number,^[^ ^14^ ^]^ swirl installation angle,^[^ ^15^ ^]^ fuel injection angle,^[^ ^16^ ^]^ fuel atomization cone angle,^[^ ^17^ ^]^ and excess air ratio^[^ ^18^ ^]^ at the combustion dome also directly affect the outlet temperature profile of the combustor. By regulating the multiphase flow coupling process at the combustion dome through the combustor structure, the temperature profile characteristics at the outlet of the combustor can be improved. However, changes in structural design often disrupt the flow in the combustor, so it is necessary to explore a more flexible combustion control method.

One recent development in combustion control^[^ ^19^ ^]^ is the use of gliding arc plasma (GAP) to improve the thermal, kinetic, and transport effects of combustion.^[^ ^20^, ^21^ ^]^ GAP‐enhanced combustion can promote fuel cracking and reforming, improve combustion efficiency, and broaden the blowout limits, especially at low temperatures, low pressures, and in lean combustion environments. Gliding arc‐enhanced combustion technology exhibits unique advantages,^[^ ^22^, ^23^ ^]^ and has therefore received widespread attention in the field of aeroengine combustion.

Numerous studies have attempted to verify the potential for gliding arc‐enhanced combustion technology to be applied in aeroengine combustors. In terms of numerical simulations, Kolev and Bogaerts^[^ ^24^ ^]^ and Bourlet et al.^[^ ^25^ ^]^ established gliding arc discharge models and simulated the breakdown and evolution of the gliding arc. In terms of engineering applications, Zhang et al. designed a gliding arc‐enhanced combustion scheme suitable for the swirl combustor of aeroengines, and conducted comprehensive research on the working characteristics,^[^ ^26^ ^]^ fuel atomization characteristics,^[^ ^27^ ^]^ and ignition characteristics.^[^ ^28^ ^]^ Feng et al.^[^ ^29^, ^30^ ^]^ proposed a multichannel gliding arc‐enhanced combustion scheme suitable for ramjets, and verified the ignition performance. In addition, Tang et al. and Sun et al.^[^ ^31^, ^32^ ^]^ and Ju et al.^[^ ^33^, ^34^ ^]^ studied the ignition characteristics of ammonia/air mixed fuels assisted by GAP, and revealed the mechanism of stable premixed swirling flames under gliding arc assistance. You et al.^[^ ^35^ ^]^ and Ombrello et al.^[^ ^36^ ^]^ analyzed the flame structure of gliding arc‐assisted methane combustion and revealed the combustion mechanism of gliding arc‐assisted low‐calorific‐value fuel. Although the gliding arc‐enhanced combustion technology has made significant progress in improving combustion efficiency and ignition characteristics, the mechanism of its influence on regulating the outlet temperature profile of the combustor has not been fully studied. In particular, there is a large gap in the literature concerning the application of GAP to aeroengine combustors.

This article considers the GAP combustion dome designed by our research team.^[^ ^28^, ^29^ ^]^ A multiphysics field diagnostic platform is constructed for the aeroengine swirl combustor, and the GAP‐assisted combustion characteristics are studied. Thermocouple measurements, particle image velocimetry (PIV), and spontaneous emission imaging are used to measure the outlet temperature field, combustor flow field, and combustion field information. The temperature profile characteristics and combustion efficiency at the outlet of the combustor are analyzed, and the mechanism whereby the gliding arc regulates the outlet temperature profile of the combustor is revealed. The results presented in this paper provide a reference for the engineering application of GAP in aeroengine combustors.

## 2 Results and Discussion

### 2.1 Effect of Gliding Arc Discharge on Combustor Flow Field

**Figure 1:**
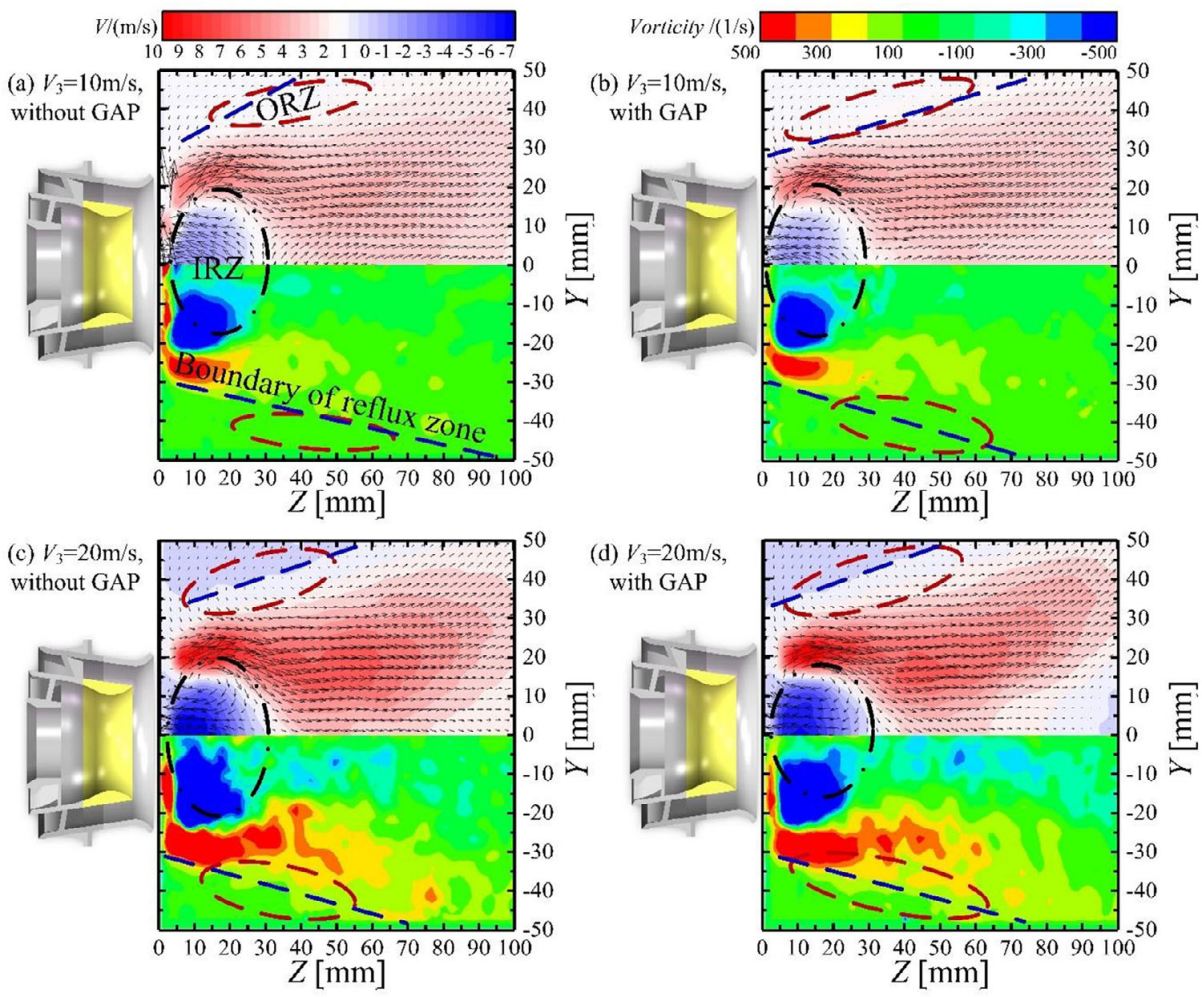
Velocity field and vorticity field of combustor.

The flow structure and vorticity field structure in the combustor determine the multiphase flow coupling process at the combustion dome, as well as the shape and combustion condition of the flame in the combustor. Thus, these structures affect the outlet temperature field profile of the combustor. This paper explores the effect of gliding arc discharge on the flow field of the combustor under the conditions of *V*_3_ = 10 and 20 m s^−1^. The results are shown in **Figure** **1**. The streamline trajectory in the upper half of each panel shows the *Z‐*direction airflow velocity and the streamlines inside the combustor. The streamlines represent the direction of airflow, and the color depth represents the airflow velocity. The lower half of each panel shows the vorticity field inside the combustor, with color depth representing the intensity of vortices. The blue dashed line represents the boundary of the recirculation zone, which is the contour line with zero velocity in the *Z*‐direction.

According to Figure 1, the flow field structure inside the combustor is a typical confined space flow field.^[^ ^37^, ^38^ ^]^ There are two main areas: the inner recirculation zone (IRZ, black oval dotted area), located downstream of the center of the combustion dome, and the outer recirculation zone (ORZ, red oval dashed area), located on the outer side. The IRZ contains the main reverse flow inside the combustor. There is a pair of vortices rotating in opposite directions downstream of the combustion dome. The outer vortex is semi‐circular with a larger *Z*‐direction scale, while the inner vortex is elliptical with a relatively small scale. Comparing Figure 1a,c, an increase in *V*_3_ enhances the range of the IRZ and ORZ, increases the velocity in the *Z*‐direction, and expands the range of vortex action in the combustor. The boundary of the recirculation zone is significantly widened as a result of the enhanced aerodynamic effect in the combustor.

Gliding arc discharge affects the flow field structure inside the combustor, especially under low‐*V*_3_ operating conditions. With *V*_3_ = 10 m s^−1^, the airflow direction at the root of the flow field IRZ (*Z* = 0–5 mm) is turbulent without gliding arc discharge and more regular with gliding arc discharge. The transport effect generated by gliding arc discharge weakens the degree of airflow turbulence.

With *V*_3_ = 10 m s^−1^, the IRZ expands in both the *Y‐* and *Z*‐directions in the case with gliding arc discharge, and the boundary of the recirculation zone widens. The temperature rise caused by gliding arc discharge leads to a local pressure increase, whereupon the IRZ expands and the boundary of the recirculation zone widens. Under *V*_3_ = 20 m s^−1^, the IRZ decreases in the *Y*‐direction when gliding arc discharge is applied and does not change significantly in the *Z*‐direction; the change in the recirculation zone is not significant. The thermal and transport effects generated by gliding arc discharge are the main factors affecting the flow field. The transport effect weakens the degree of airflow turbulence, but has a certain contraction effect on the flow field. The thermal effects cause a local increase in air pressure, which is manifested as an expansion of the flow field. An increase in *V*_3_ enhances the cooling effect of the airflow on the arc, and the transport effect dominates the contraction of the flow field. Therefore, under a larger *V*_3_, the influence of the gliding arc on the flow field becomes less significant, and there is even a trend of decreasing IRZ.

The overall analysis shows that gliding arc discharge reduces the range of action of vortices at 400–500 s^−1^ and from −400 to −500 s^−1^, but the range of action of the overall vorticity field will increase, especially in the *Z*‐direction. This means that gliding arc discharge enhances the aerodynamic effect inside the combustor, and enhances the symmetry of the downstream recirculation zone of the combustor about *Y* = 0, which is conducive to improving the uniformity of the outlet temperature.

### 2.2 Distribution and Influencing Factors of Combustor Outlet Temperature Field

#### 2.2.1 Outlet Temperature Distribution of Combustor

**Figure 2:**
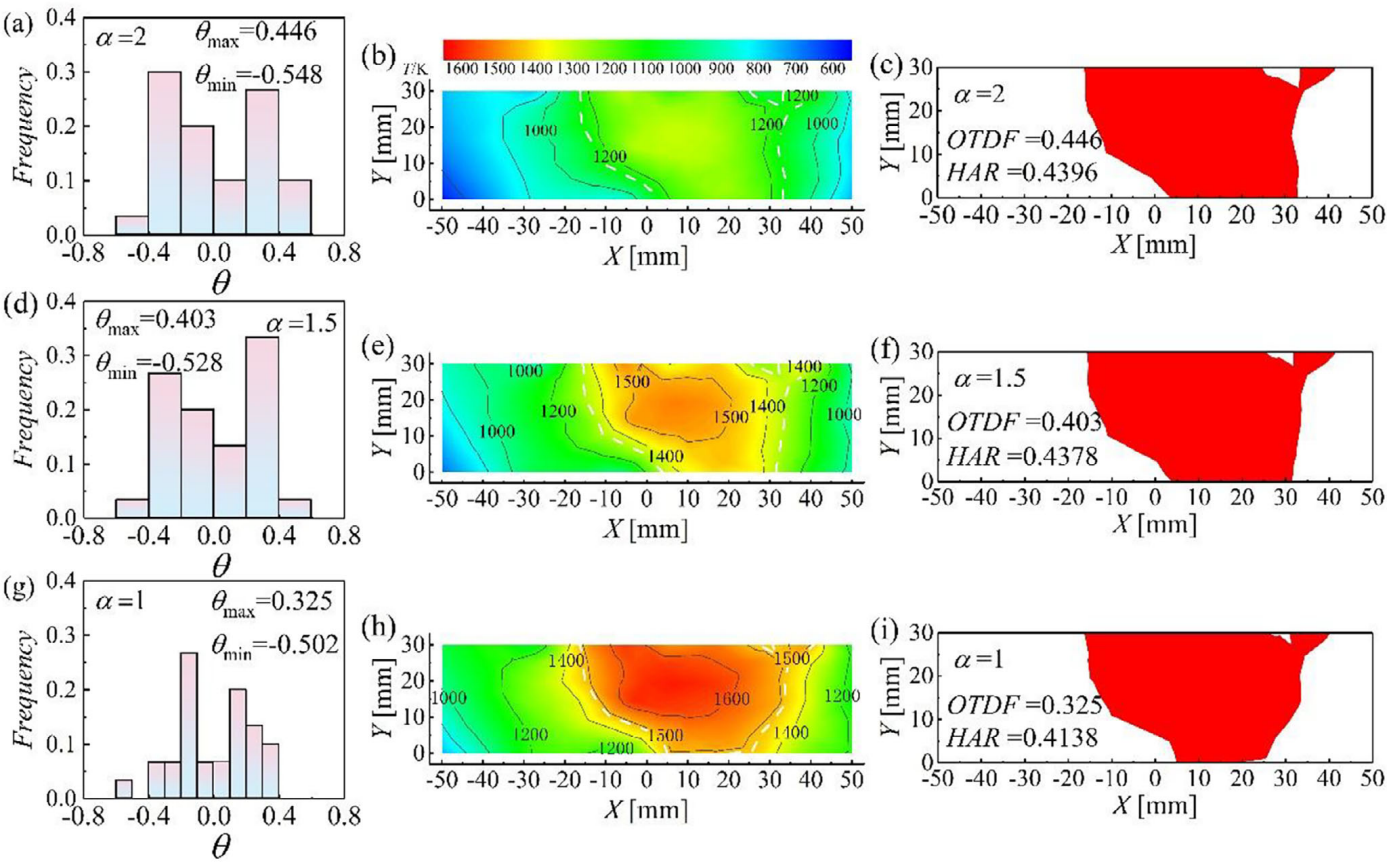
Outlet temperature distribution characteristics of combustor.

**Figure** **2** shows the outlet temperature field profile of the combustor under the conditions of *V*_3_ = 15 m s^−1^, *α* = 1, 1.5, 2, and no gliding arc‐assisted combustion. Figure 2a,d,g shows the frequency profile of the outlet temperature factor of the combustor. Figure 2b,e,h shows cloud charts of the outlet temperature distribution of the combustor. The white dashed lines represent the outer contour of the hot spot area. Figure 2c,f,i shows the outlet profile area of the °ks in the combustor. In Figure 2b,e,h, a clear high‐temperature zone appears in the outlet area downstream of the combustion dome. This high‐temperature zone is centered, and the surrounding temperature, especially near the outlet wall, is relatively low. This is the result of the cooling airflow on the flame tube wall, which conforms to the outlet temperature distribution law of swirl combustors.^[^ ^39^ ^]^

The variation in the excess air coefficient has a significant impact on the outlet temperature profile characteristics of the combustor. When *α* = 2, Figure 2b indicates that the outlet temperature of the combustor is low and there is no temperature zone hotter than 1400 K. According to Figure 2a,c, *θ*_max_ = 0.446, *θ*_min_ = −0.548 under this condition, and the main frequency distribution range of θ is from −0.4 to −0.2, with a hot‐streak area ratio (HAR) of 0.4396 and an outlet temperature distribution factor (OTDF) of 0.446. The differences in the outlet temperature of the combustor and the proportion of the hot‐streak area are relatively large, and the combustor exit temperature has poor uniformity. As *α* decreases, the fuel–air ratio in the combustor becomes closer to the stoichiometric ratio. As shown in Figure 2b,e,h, when *α* decreases from 2 to 1, a 1500 K temperature zone and a 1600 K high‐temperature zone are observed at the outlet of the combustor. Simultaneously, the hot‐streak area and OTDF at the outlet of the combustor decrease (from HAR = 0.4396 to HAR = 0.4138 and from OTDF = 0.446 to OTDF = 0.325), *θ*_max_ decreases from 0.446 to 0.325, and *θ*_min_ increases from −0.548 to −0.502. The distribution range of *θ* becomes more concentrated. This indicates that as the combustor transitions from lean fuel conditions to chemically appropriate conditions, the outlet temperature of the combustor increases and the variation in outlet temperature decreases. This is because the air in the combustor at lean fuel conditions is greater than the air flow required for fuel combustion, and air that is not involved in combustion cools the flame, so the combustor exit temperature at lean fuel conditions is low.

#### 2.2.2 Characteristics of Outlet Temperature Profile in GAP‐Assisted Combustor

**Figure 3:**
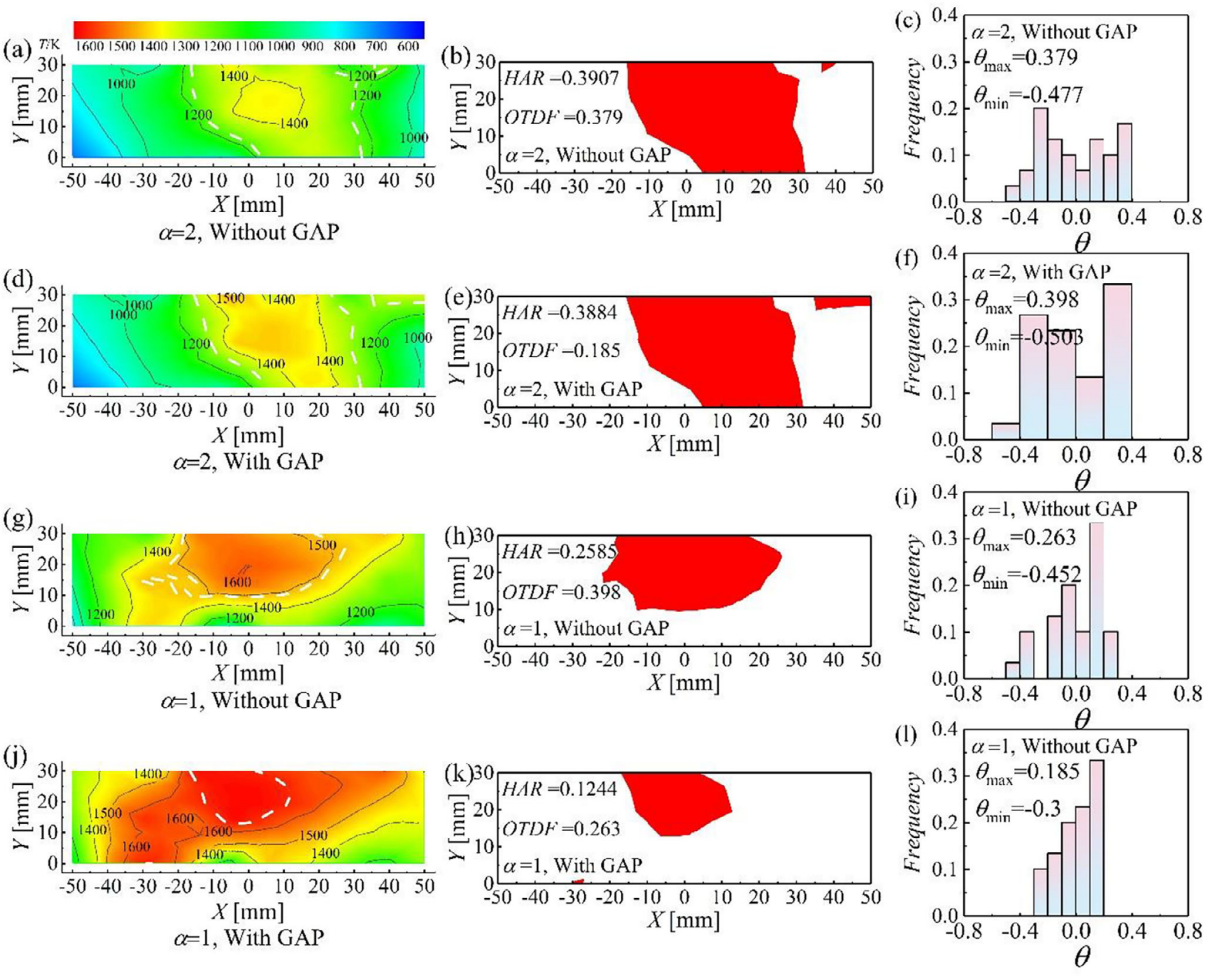
Outlet temperature profile characteristics of the combustor under gliding arc plasma‐assisted combustion conditions.

The influence of GAP‐assisted combustion on the outlet temperature profile characteristics of the combustor is analyzed under *V*_3_ = 20 m s^−1^ and excess air coefficients of 1 and 2. **Figure** **3**a,d,g,i shows cloud charts of the combustor outlet temperature, Figure 3b,e,h,k displays HAR at the combustor outlet, and Figure 3c,f,i,l shows the frequency profile of the outlet temperature factor of the combustor. GAP‐assisted combustion improves the outlet temperature profile of the combustor. Taking *α* = 2 as an example, regions with temperatures exceeding 1400 K are confined to small areas near the central region of the combustor outlet without GAP assistance, whereas regions with temperatures exceeding 1400 K are significantly larger under GAP‐assisted combustion. This indicates that GAP enhances the expansion of high‐temperature zones at the combustor outlet. This phenomenon is more pronounced when *α* = 1. Indeed, large areas in which the temperature exceeds 1500 K and 1600 K are observed at the combustor outlet. Previous studies have shown that gliding arc discharge generates active particles and high‐energy electrons (with an electron density of ≈5.5 × 10^23^ m^−3^), which collide with kerosene molecules during their motion ($e+C_{12}H_{26}\overset{impact}{\to }C_{x}H_{y}\left(x<12,y<26\right)$). The carbon chains between kerosene molecules break up, forming small molecules with low‐carbon chains, which reduces the viscosity of the fuel droplets and results in more uniform atomization. In addition, the *N*_2_(*C* ^3^Π_u_ → *B* ^3^Π_g_) ($e+N_{2}\left(X^{1}\Sigma_{g}^{+}\right)\to N_{2}\left(C^{3}\Pi_{u}\right)+e,\Delta e=13.06eV$, *N*_2_(*C* ^3^Π_u_) → *N*_2_(*B* ^3^Π_g_) + *hv*), $N_{2}^{+}\left(B^{2}\Sigma_{u}^{+}\to X^{2}\Sigma_{g}^{+}\right)$ ($e+N_{2}\left(X_{1}\Sigma_{g}^{+}\right)\to N_{2}\left(B^{2}\Sigma_{u}^{+}\right)+2e\Delta E$, $e+N_{2}\left(X_{2}\Sigma_{g}^{+}\right)\to N_{2}\left(B^{2}\Sigma_{u}^{+}\right)+e$, $N_{2}\left(B^{2}\Sigma_{u}^{+}\right)\to N_{2}\left(X^{2}\Sigma_{g}^{+}\right)+hv$), and $N_{2}^{+}\left(B^{2}\Sigma_{u}^{+}\to X^{2}\Sigma_{g}^{+}\right)$ ($e+N_{2}\left(X^{1}\Sigma_{g}^{+}\right)\to N_{2}\left(B^{2}\Sigma_{u}^{+}\right)+2e,\Delta e=18.76eV$, $e+N_{2}^{+}\left(X^{2}\Sigma_{g}^{+}\right)\to N_{2}^{+}\left(B^{2}\Sigma_{u}^{+}\right)+e,\Delta e=3.13eV$, $N_{2}^{+}\left(B^{2}\Sigma_{u}^{+}\right)\to N_{2}^{+}\left(X^{2}\Sigma_{g}^{+}\right)$) generated by gliding arc discharge have a relatively high kinetic activity. Active particles such as O (*e* + *O*_2_ → *O* + *O*(1*D*) + *e*Δ*E*, *e* + *O*_2_ → *O* + *O*(1*S*) + *e*Δ*E*) and OH play a role in promoting combustion in the combustion reaction. Consequently, more complete combustion heat release occurs, and the temperature rise at the combustor outlet is significantly increased.

GAP‐assisted combustion expands the spatial extent of high‐temperature zones at the combustor outlet, but the hot‐streak area does not increase, and OTDF has a decreasing trend. As evidenced by Figure 3, with *α* = 2, HAR without GAP assistance is 0.3907 and OTDF = 0.379, with *θ*_max_ = 0.379 and *θ*_min_ = −0.477. With GAP actuation, HAR decreases slightly to 0.3884, OTDF decreases to 0.185 (a reduction of more than 51%), accompanied by *θ*_max_ = 0.398 and *θ*_min_ = −0.503. HAR is slightly reduced after applying GAP‐assisted combustion, and the chamber exit temperature uniformity is significantly improved. The maximum value of *θ* tends to increase, although its distribution becomes more spatially concentrated, which explains why the change in HAR is not pronounced. As evidenced by Figure 3h,k, with *α* = 1, the reduction in HAR and OTDF with GAP assistance is significantly more pronounced. Without plasma actuation, HAR = 0.2585 and OTDF = 0.398, whereas with GAP intervention, HAR decreases sharply to 0.1244 and OTDF decreases to 0.263, a reduction of more than 51%. Additionally, the outlet temperature distribution under plasma‐assisted combustion is more concentrated. GAP discharge improves fuel atomization effect (as evidenced by Figure 1), and the plasma discharge expands the vorticity field scale, enlarging the spatial distribution of fuel within the combustor. This broadens the fuel‐derived heat release zones and suppresses localized combustion phenomena, resulting in a more uniform outlet temperature profile at the combustor.

### 2.3 Outlet Temperature Profile Uniformity Analysis

#### 2.3.1 STDF and its Influencing Factors

**Figure 4:**
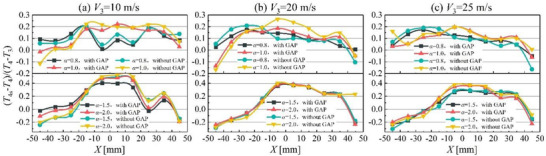
Spanwise temperature distribution factor variations.

Cloud charts of the temperature distribution at the combustor exit provide a macroscopic analysis of the temperature characteristics, but do not enable a quantitative evaluation of the exit temperature field. The spanwise temperature distribution factor (STDF) characterizes the difference in spanwise temperature of the combustor outlet. Therefore, STDF is introduced to allow a quantitative assessment of the combustor exit temperature fields. The influence of factors such as GAP‐assisted combustion, combustor inlet flow velocity, and excess air coefficient on STDF at the combustor outlet are illustrated in **Figure** **4**. Under different combustion dome inlet flow velocity conditions, the variation patterns are generally similar. The trends can be broadly categorized into two types. The first type corresponds to the STDF variation curve under the operating condition with *α* = 0.8, exhibiting a wavelike variation trend. This curve typically shows 2–3 peaks, particularly within the range of *X* = −20–20 mm, where the STDF fluctuations are significantly more intense. This phenomenon is attributed to the expansion and extension of the high‐temperature zone in the outlet temperature profile of the combustion. The second type corresponds to the STDF variation curves under *α* = 1.0, 1.5, 2.0, which exhibit multi‐peaked convex profiles, typically showing 1–2 distinct peaks. Within the *X* = −20–20 mm region, the STDF values are significantly higher, indicating more pronounced spanwise temperature fluctuations at the combustor exit.

An increase in the excess air coefficient leads to an expansion of the STDF fluctuation range. This trend is consistently observed across different combustor inlet flow velocity conditions. Taking *V*_3_ = 10 m s^−1^ as an example, when *α* = 0.8, STDF fluctuates within the range of 0–0.2. When *α* = 1.0, the STDF fluctuation range expands to −0.1–0.25, and further broadens to −0.2–0.45 at *α* = 1.5. This significant expansion of the STDF fluctuation range indicates that higher excess air coefficients intensify the spanwise temperature variations at the combustor exit.

GAP‐assisted combustion reduces the fluctuation range of STDF at the combustor exit. This suppression effect is particularly pronounced within the region from *X* = −20–20 mm, in which range STDF with GAP‐assisted combustion is basically smaller than that without GAP‐assisted combustion. Under the operating condition of *V*_3_ = 25 m s^−1^ and *α* = 0.8, the STDF fluctuation range is −1.8–0.2 without GAP assistance, narrowing to −0.8–0.17 with GAP‐assisted combustion. This is not an isolated case, as demonstrated across multiple experimental trials. To characterize the fluctuations in STDF, the variance of the curve within the range of *X* = −20–20 mm is presented in **Table** **1**.

**Table 1:** Variance of STDF.

*V*_3_ *α*	10 m s^−1^		20 m s^−1^		25 m s^−1^	
	Without GAP	With GAP	Without GAP	With GAP	Without GAP	With GAP
0.8	0.1346	0.1350	0.1563	0.1636	0.1573	0.1529
1.0	0.1665	0.1618	0.1582	0.1272	0.1050	0.0967
1.5	0.2577	0.2336	0.1859	0.1768	0.1281	0.1217
2.0	0.2869	0.2713	0.1994	0.1753	0.1836	0.1567

Comparative analysis reveals that, as *α* increases, the variance of STDF generally exhibits an increasing trend under *V*_3_ = 10 and 20 m s^−1^. However, for *V*_3_ = 25 m s^−1^, the STDF variance initially decreases and then increases. Overall, STDF exhibits greater variance under *α* = 2.0. This phenomenon is attributed to the cooling effect of unreacted air on the flame when *α* = 2.0, resulting in increased spatial discreteness of the outlet temperature profile.

The influence of *V*_3_ on the variance of STDF exhibits two diametrically opposite trends. When *α* = 0.8, the STDF variance increases with rising *V*_3_, whereas for *α* = 1.0, 1.5, and 2.0, the STDF variance demonstrates a decreasing trend with higher *V*_3_. As illustrated in Figure 1, an increase in *V*_3_ enhances the aerodynamic interactions within the combustor, improves the fuel atomization effect, and extends the effective mixing zone of the fuel–air mixture. Consequently, under both stoichiometric and lean operating conditions, a higher *V*_3_ reduces the spatial discreteness of the outlet temperature profile. Under fuel‐rich operating conditions, the pyrolysis of unburned fuel consumes thermal energy from the flame. This process exhibits greater spatial randomness within the combustor, resulting in enhanced variability of the outlet temperature profile under fuel‐rich conditions.

Regarding the impact of GAP‐assisted combustion on STDF, except when *V*_3_ = 10 m s^−1^ with *α* = 0.8 and 20 m s^−1^ with *α* = 0.8, the STDF variance under GAP‐assisted combustion conditions is less than that without GAP‐assisted combustion. This indicates that the application of GAP‐assisted combustion can significantly reduce the STDF fluctuation and improve the temperature distribution uniformity in the spanwise direction.

#### 2.3.2 OTDF and HAR at Combustion Exit

**Figure 5:**
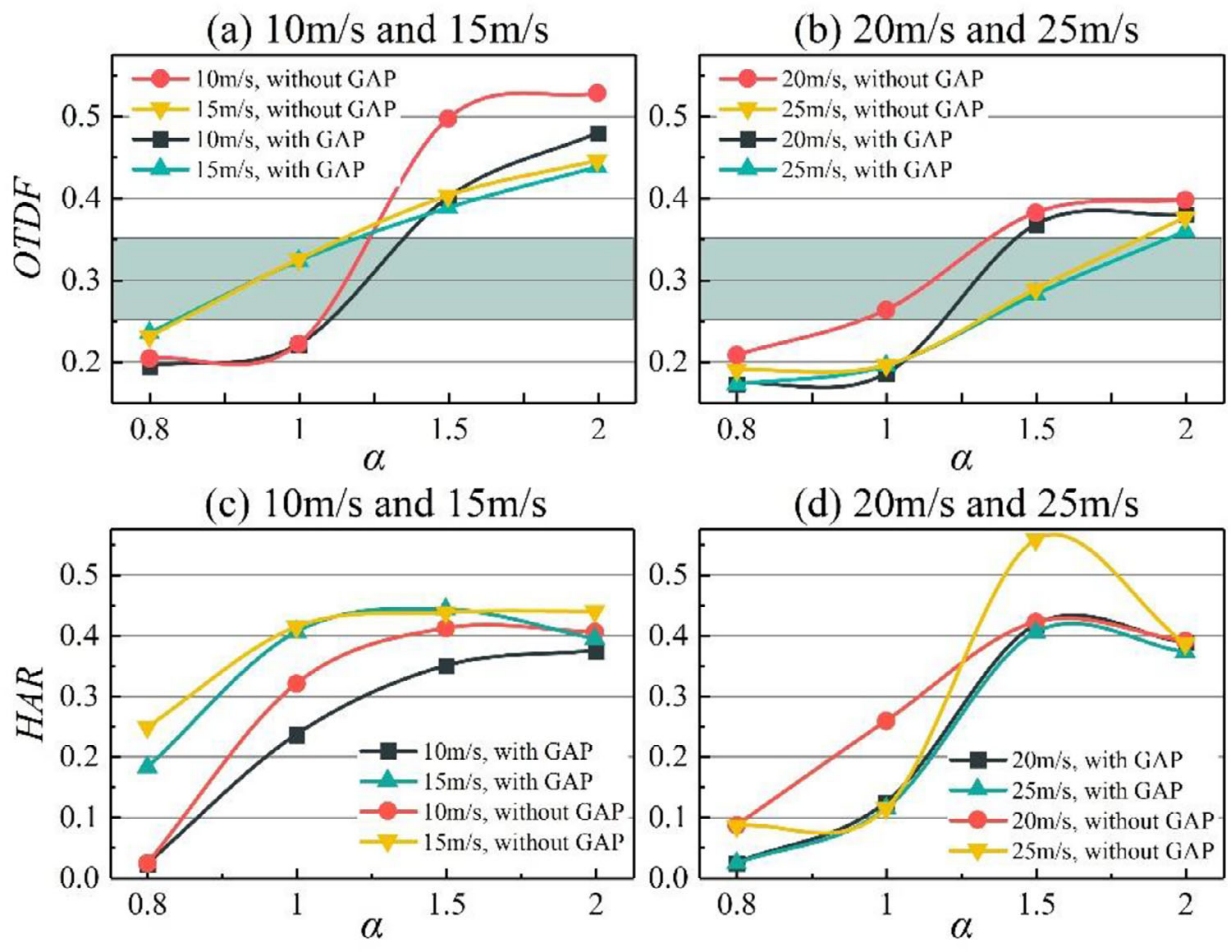
Variation of outlet temperature distribution factor and hot‐streak area ratio.

The combustor outlet temperature and hot streaks are critical factors influencing the durability and lifespan of turbine blades. Optimizing the combustor outlet temperature profile and controlling HAR in the outlet temperature distribution significantly impact the performance of aeroengines and gas turbines. The influence of *V*_3_, *α*, and GAP excitation on OTDF and HAR are now investigated. The light‐green boxes in **Figure** **5**a,b demarcate the theoretically acceptable range for OTDF.

As *α* increases from 0.8 to 2.0, both OTDF and HAR generally exhibit increasing trends, with the exception of *V*_3_ = 25 m s^−1^ without GAP assistance. When *V*_3_ = 10 m s^−1^ with *α* = 2.0 and *V*_3_ = 15 m s^−1^ with *α* = 1.5 and 2.0, OTDF deviates greatly from the normal range (0.25–0.35), while generally stay around the normal range in other conditions. Fuel–air mixing is a critical factor influencing the combustor outlet temperature profile. Under lean operating conditions, the reduced fuel concentration combined with contracted recirculation zone boundaries in the combustor (as shown in Figure 1 for low‐flow conditions) restricts the effective fuel dispersion area. This spatial limitation intensifies the localized heat release. The HAR variation trends with respect to *α* in Figure 5c,d further validate this mechanism.

Macroscopically, OTDF decreases with increasing *V*_3_. Under *V*_3_ = 10 m s^−1^, OTDF varies from 0.2–0.55 with large fluctuations, and deviates from the normal range. Under *V*_3_ = 15 m s^−1^, OTDF only varies from 0.23–0.45, and when *V*_3_ reaches 25 m s^−1^, the variation range of OTDF is reduced to 0.18–0.35. The influence of *V*_3_ on HAR is particularly obvious under the two working conditions of *α* = 0.8 and *α* = 1.0, where HAR increases with increasing *V*_3_; the influence of *V*_3_ on HAR is not obvious under other working conditions. Lefebvre et al.^[^ ^40^ ^]^ identified the combustor length and the mixing of combustion products as two critical factors influencing OTDF. An increase in the combustor inlet flow velocity expands the recirculation zone within the combustor, thereby enlarging the effective fuel reaction area. Additionally, higher inlet flow velocities enhance the turbulence intensity in the airflow regulation zone, which accelerates the fuel–air mixing process. This enhanced mixing results in a more uniform outlet temperature profile.

GAP‐assisted combustion contributes to improved outlet temperature profile characteristics and reduced HAR at the combustor exit. This phenomenon is particularly pronounced under *V*_3_ = 10 m s^−1^ with *α* = 1.5. Under these conditions, OTDF without GAP‐assisted combustion is 0.4971 and HAR = 0.4116; with GAP‐assisted combustion, OTDF is 0.4006 and HAR = 0.3499. Thus, GAP excitation reduces OTDF by 19.41% and HAR by 14.99%. GAP‐assisted combustion can reduce OTDF by up to 29.56% (with *V*_3_ = 20 m s^−1^, *α* = 1.5) and HAR by up to 72.6% (with *V*_3_ = 25 m s^−1^, *α* = 0.8). Gliding arc discharge improves the fuel atomization quality through the temperature rise effect, high‐energy electron collision, and the enhanced aerodynamics in the combustor. At the same time, the GAP discharge widens the scale of the vorticity field in the combustor, which increases the fuel action area. Thus, the uniformity of the temperature field at the outlet of the combustor is significantly improved.

### 2.4 Combustion Efficiency and Outlet Temperature Rise

**Figure 6:**
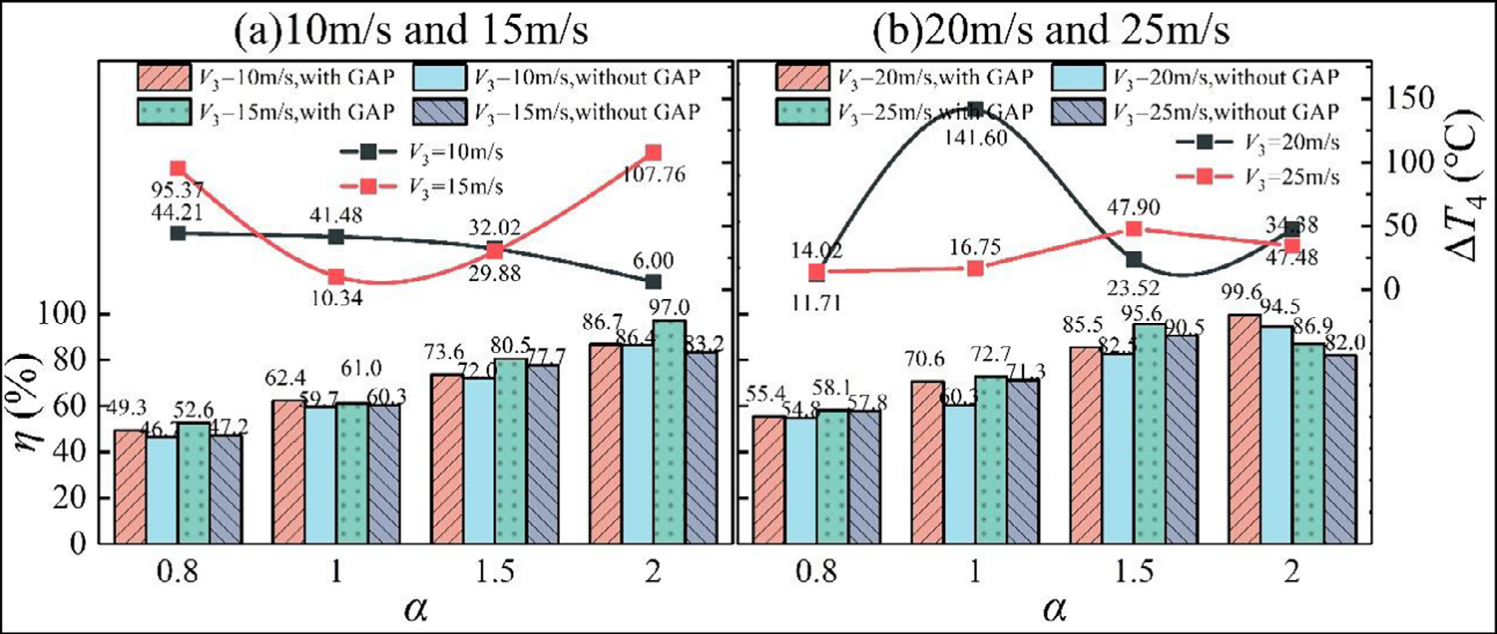
Combustion efficiency and temperature rise at combustor outlet.

The combustion efficiency of the combustor and the temperature increment at the outlet of the combustor under different working conditions are calculated using the enthalpy increase method. The results are shown in **Figure** **6**, where the histograms show the combustion efficiency and the dotted lines show the temperature increment at the outlet of the combustor under GAP‐assisted combustion conditions compared with no GAP‐assisted combustion (Δ*T*_4_ = *T*_4,withGAP_ − *T*_4,withoutGAP_, where *T*_4,withGAP_ represents the average outlet temperature of the combustor with GAP‐assisted combustion and *T*_4,withGAP_ represents the average outlet temperature of the combustor without GAP‐assisted combustion). Regardless of whether GAP‐assisted combustion is applied, the kerosene combustion is more complete under lean oil conditions. With *V*_3_ = 10 m s^−1^ in the case of GAP‐assisted combustion, as *α* increases from 0.8 to 2, the combustion efficiency increases from 49.3% to 86.7% because there is more oxygen in the combustor under the lean oil condition than required for complete combustion of the fuel. The fuel burns more fully and the chemical energy is fully released. The combustion efficiency in the combustor increases with increasing *V*_3_. Figure 1 shows that an increase in *V*_3_ enhances the forward and reverse airflow velocity in the combustor, which increases the vortex intensity, aerodynamic effect, and fuel atomization. This leads to improved heat and mass exchange rates in the combustion process, which accelerates the combustion speed and improves the combustion efficiency.

For large‐molecule hydrocarbon fuels, the high‐energy electrons generated by gliding arc discharge first collide with kerosene molecules, splitting them into small‐molecule fuels such as H_2_, CH_4_, CH_2_O, and C_2_H_4_. This reduces the *Le* ^*^ of the combustion process and increases the flame temperature (*U* ^2^ *lnU* ^2^ = −β*H + β*Q* − β′(Le* − 1)Ka*). Under the action of high‐energy particle clusters in gliding arcs, some free radicals and new reaction pathways (**Table** **2**, R(1)–R(12)) are then generated to reduce the activation energy of combustion reactions. The Joule heat generated by gliding arc discharge is added to the combustion reaction, accelerating the flame propagation and combustion process (Table 2; the R(13) path is more sensitive). The final gliding arc discharge changes the flow field structure during the combustion process, and modifies the flame stretch to Ka*. This effect depends on Le*. Improving the local flow field through gliding arcs improves the mixing between combustion products and unburned mixtures, thereby enhancing the heat release rate of the flame and resulting in more complete combustion.^[^ ^20^ ^]^

**Table 2:** Key reaction pathways of plasma‐assisted combustion.^[^ ^20^ ^]^

Chain‐initiation	Number
*e* + *O*_2_ = *O* + *O*(^1^ *D*)	R(1a)
$e+O_{2}^{+}=O+O$	R(1b)
$N_{2}\left(A,B,C\right)+O_{2}=O+O+N_{2}$	R(2a)
$N^{+}+O=O^{+}+NO$	R(2b)
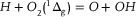	R(3)
$O_{3}+O_{2}=O+O_{2}+O_{2}$	R(4)
*O*(^1^ *D*) + *H*_2_ = *OH* + *O*	R(5)
$NO+HO_{2}=NO_{2}+HO$	R(6)
$H+NO_{2}=NO+OH$	R(7)
$HO_{2}+HO_{2}=H_{2}O_{2}$	R(8)
*e* + *RH* = *R* + *H* + *e*	R(9)
$e+RH=R^{\prime }+H+CH_{3}+e$	R(10)
*e* + *RH* = *R* ^+^ + 2*e* + 2*H*	R(11)
$N_{2}^{*}+RH=R+H+N_{2}$	R(12)
$H+O_{2}=OH+O$	R(13)

GAP‐assisted combustion improves the combustion efficiency and outlet temperature rise of the combustor. Under *V*_3_ = 15 m s^−1^ and *α* = 2, the application of GAP‐assisted combustion increases the combustion efficiency by 13.8% and enhances the temperature rise at the outlet of the combustor by 107.76°C. With *V*_3_ = 20 m s^−1^ and *α* = 1, the combustion efficiency increases by 10.3% and the temperature rise at the outlet of the combustor increases by 141.6°C. Gliding arc discharge improves the fuel atomization. The active particles produced by gliding arc discharge play an auxiliary role in combustion, changing the combustion reaction path and making the combustion heat release more complete. Thus, the temperature rise at the outlet of the combustor increases and the combustion efficiency improves.

**Figure 7:**
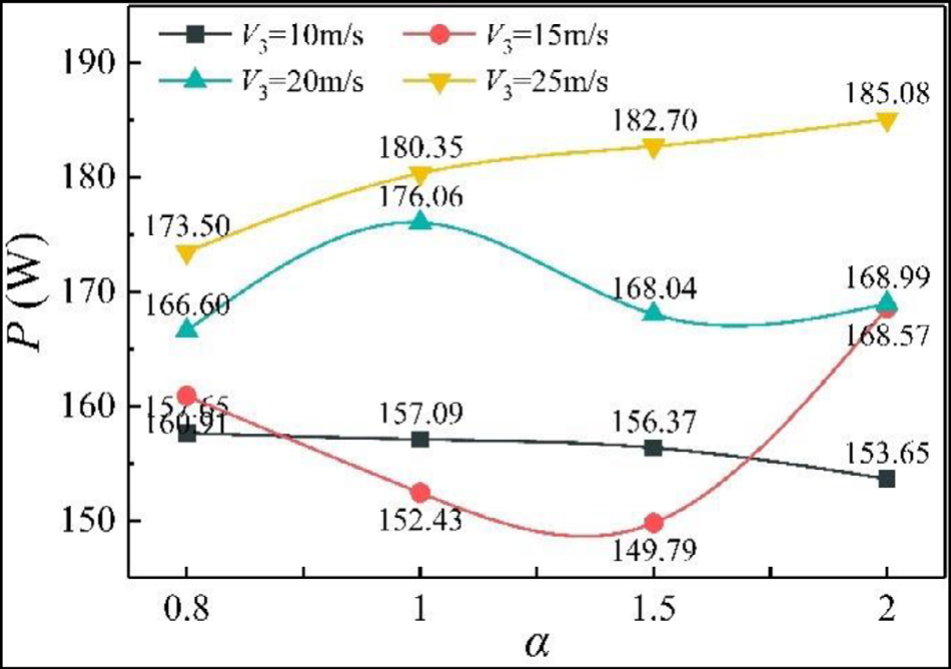
Gliding arc discharge power.

**Figure** **7** shows the gliding arc discharge power, which is a symbol of the processing ability of the gliding arc. The Joule heat of the current is an important source of the temperature rise effect of the gliding arc discharge. In addition, the gliding arc discharge power directly affects the production of active particles.^[^ ^27^ ^]^ The figure shows that the discharge power of the gliding arc is affected by *V*_3_ and *α*. This is because the development of the gliding arc depends on the airflow driving action. The breakdown difficulty of the ambient medium also influences the discharge power of the gliding arc. Analysis shows that the variation in gliding arc discharge power with respect to *α* is roughly the same as that of Δ*T*_4_. A higher gliding arc discharge power increases the outlet temperature of the combustor. Under a higher gliding arc discharge power, the gliding arc generates more Joule heat, the temperature rise effect is more obvious, and the number of active particles excited between the cathode and anode will increase. The overall effect is to enhance the combustion support.

### 2.5 Flame Shape in Combustor with Gliding Arc Assistance

**Figure 8:**
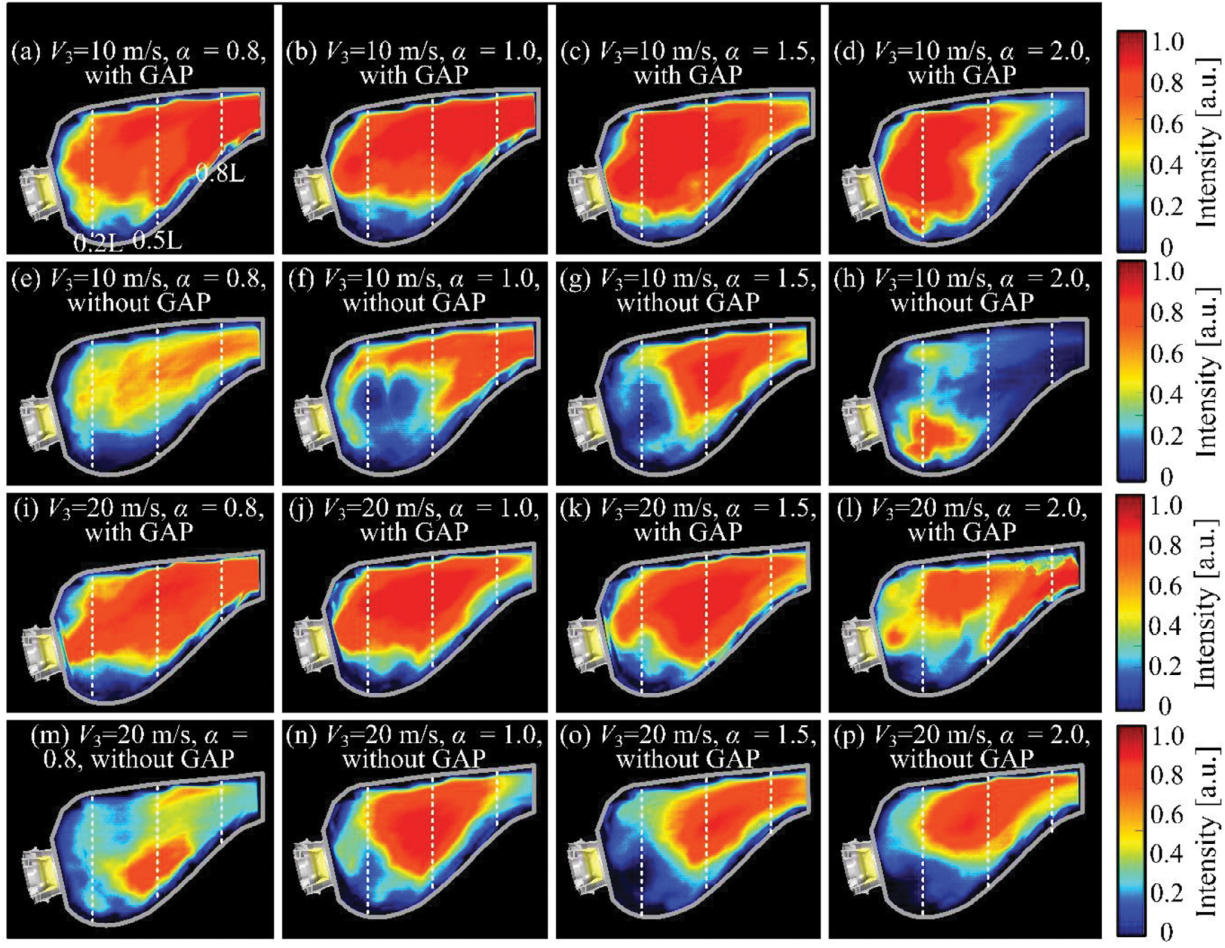
Flame structure in combustor under different conditions.

The previous analysis shows that gliding arc excitation, *V*_3_, and *α* have significant effects on the uniformity of the outlet temperature profile. These differences are caused by changes in the basic flow field structure in the combustor, which modify the flame structure. By analyzing the flame structure in the combustor, the mechanism whereby the gliding arc influences the outlet temperature profile in the combustor is now investigated. **Figure** **8** shows time‐averaged flame images of the C_2_^*^ group under different conditions, as obtained from 500 flame images in the process of stable combustion. The red area in the figure denotes the most intense heat release, indicating the core area of the combustion reaction.

The flame under GAP‐assisted combustion is a typical attached swirling flame. It is close to the outlet of the swirler and is attached to the combustion dome. The core area of the combustion reaction is relatively concentrated. Without GAP‐assisted combustion, the flame is a raised swirling flame, which is separated from the combustion dome and resides in the recirculation zone downstream of the combustor. The core area of the combustion reaction is mostly discrete.

Figure 5 indicates that the uniformity of the combustor outlet is optimal under GAP‐assisted combustion with *α* = 1.0 or *α* = 1.5 and a high flow rate. The combustor can be divided into three typical flame residence zones according to its characteristic length *L*: 0.2*L*, 0.5*L*, and 0.8*L*. Comprehensive analysis of Figure 5 and Figure 8 indicates that the uniformity of the outlet temperature of the combustor is improved when the combustion reaction core is farther forward (near 0.2*L*) and has a larger area. In this scenario, the flame location is closer to the head of the combustor and the fuel residence time is increased, ensuring a greater combustion heat release. This results in a more uniform outlet temperature profile and the earlier the core area of the combustion reaction is, the higher the combustion efficiency. The combustor flame under GAP‐assisted combustion basically resides before 0.2*L*, whereas that without GAP‐assisted combustion is located from 0.5*L*–0.8*L*. The combustion reaction core area is smaller in the absence of GAP‐assisted combustion. Therefore, GAP‐assisted combustion makes the temperature distribution of the combustor outlet more uniform and enhances the combustion efficiency.

### 2.6 Mechanism of Gliding Arc Modulating Combustor Outlet Temperature

**Figure 9:**
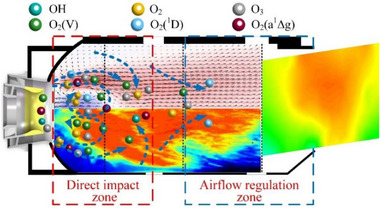
Effect mechanism of GAP‐assisted combustion on outlet temperature of combustor.

The above analysis shows that the resident flame at the dome affects the outlet temperature profile and temperature rise of the combustor. Therefore, this section summarizes the influence of GAP‐assisted combustion on the outlet temperature profile of the combustor. As shown in **Figure** **9**, the combustor is divided into two areas: a direct impact zone and an airflow regulation zone. The direct impact zone is the region on which the arc directly acts, and contains most of the active particles and energy generated by gliding arc discharge. The airflow regulation zone is subject to the direct impact of the airflow in the mixing hole, and supplementing the airflow will affect the mixing and dilution of the flame.

In terms of the combustion efficiency and chamber outlet temperature increment, the influence mechanism of the gliding arc can be attributed to three aspects. First, the effect of the flame's position moving forward. According to Figure 8, gliding arc‐assisted combustion allows the combustion chamber flame to remain near the dome of the combustor, and the combustion zone moves forward, allowing the combustor flame to have greater development space and longer reaction time. The combustion reaction proceeds more thoroughly and the heat release is more complete. Second, the auxiliary combustion effect of active particles. During the sliding arc discharge process, numerous active particles (OH, O, O_2_) are generated.^[^ ^20^ ^]^ These active particles participate in the combustion reaction and play a role in assisting combustion, accelerating the process of the combustion reaction and making combustion more thorough. Finally, the effect of gliding arc discharge energy injection and temperature rise on the activation energy of combustion reaction. During the gliding arc discharge process, a large amount of energy is continuously input into the combustor, and this energy changes the physical properties of the fuel in the form of high‐speed moving active particles and temperature rise effects, and reduces the activation energy of combustion reaction (Arrhenius’ law: *k*(*T*) = *AT^b^* exp (− *E_a_*/*RT*), where *E*_a_ is the activation energy and *T* is the temperature). The combustion reaction rate increases, and the combustion heat release is more complete. In addition, the sliding arc promotes the combustion reaction by enhancing the combustor flow field structure and oil mist. The above reasons combine in the combustion process, resulting in a higher outlet temperature and better combustion efficiency of gliding arc‐assisted combustion.

The flame residence position is the key factor influencing the uniformity of the combustor outlet temperature. Gliding arc‐assisted combustion allows the combustion chamber flame to remain near the dome of the combustor. The closer the combustion heat release zone is to the dome of the combustion chamber, the better the flame can develop in the combustion chamber, allowing combustion heat release to occur over a larger space and improving the local low/high temperature area. Moreover, after the flame passes through the airflow regulation zone, the mixing, dilution, and regulation of the airflow allow the flame to develop more fully, so GAP‐assisted combustion improves the temperature uniformity at the outlet of the combustor. In addition, gliding arc discharge promotes the flow field and fuel atomization, which enhance the uniformity of the outlet temperature profile.

## 3 Conclusion

To verify the effectiveness of GAP‐assisted combustion in improving the outlet temperature profile of a combustor, this study developed a gliding arc‐assisted combustion experimental system. The temperature at the exit of the combustor and the flame in the combustion process were collected by thermocouple measurements and spontaneous emission imaging. The temperature distribution characteristics and combustion efficiency at the exit of the combustor were comprehensively analyzed. The main conclusions are as follows:

1) The temperature rise effect of gliding arc discharge expands the flow field, while the transport effect reduces the degree of airflow turbulence and shrinks the flow field. The temperature rise and transport effects are relatively balanced under the action of the airflow. Increasing *V*_3_ enhances the aerodynamic effect in the combustor and increases the range of IRZ and ORZ, the flow velocity in the *Z*‐direction, and the scale of vortices in the combustor. The boundary of the recirculation zone is also widened. After gliding arc discharge is applied, the size of the vortices decreases in the range of 300–500 s^−1^ and from −300 to −500 s^−1^, but the size of the overall vortices increases, especially in the *Z*‐direction. Larger vortices enhance the scope of the combustion effect, having a positive effect on the combustion efficiency and outlet temperature profile.
2) The flame residence position and the active particles produced by gliding arc discharge are key factors affecting the combustion efficiency and outlet temperature profile. Under the action of gliding arc discharge, the flame is close to the combustion dome and resides in the direct impact zone. Gliding arc discharge results in a more complete combustion process and greater combustion heat release. Moreover, the airflow regulation zone provides space and unburned air for the full development of the flame, thereby improving the temperature rise of the combustor and the outlet temperature profile.
3) GAP‐assisted combustion has a significant effect on improving the combustion efficiency and temperature distribution at the outlet. Under the conditions of *V*_3_ = 20 m s^−1^ and *α* = 1, the combustion efficiency increases by 10.3% and the temperature rise at the outlet of the combustor increases by 141.6°C. Under the conditions of *V*_3_ = 20 m s^−1^ and *α* = 1.5, GAP‐assisted combustion reduces OTDF by 29.56%, and under the conditions of *V*_3_ = 25 m s^−1^ and *α* = 0.8, GAP‐assisted combustion reduces HAR by 72.6%.

## 4 Experimental Section

### Experimental Platform and Combustor Model

**Figure 10:**
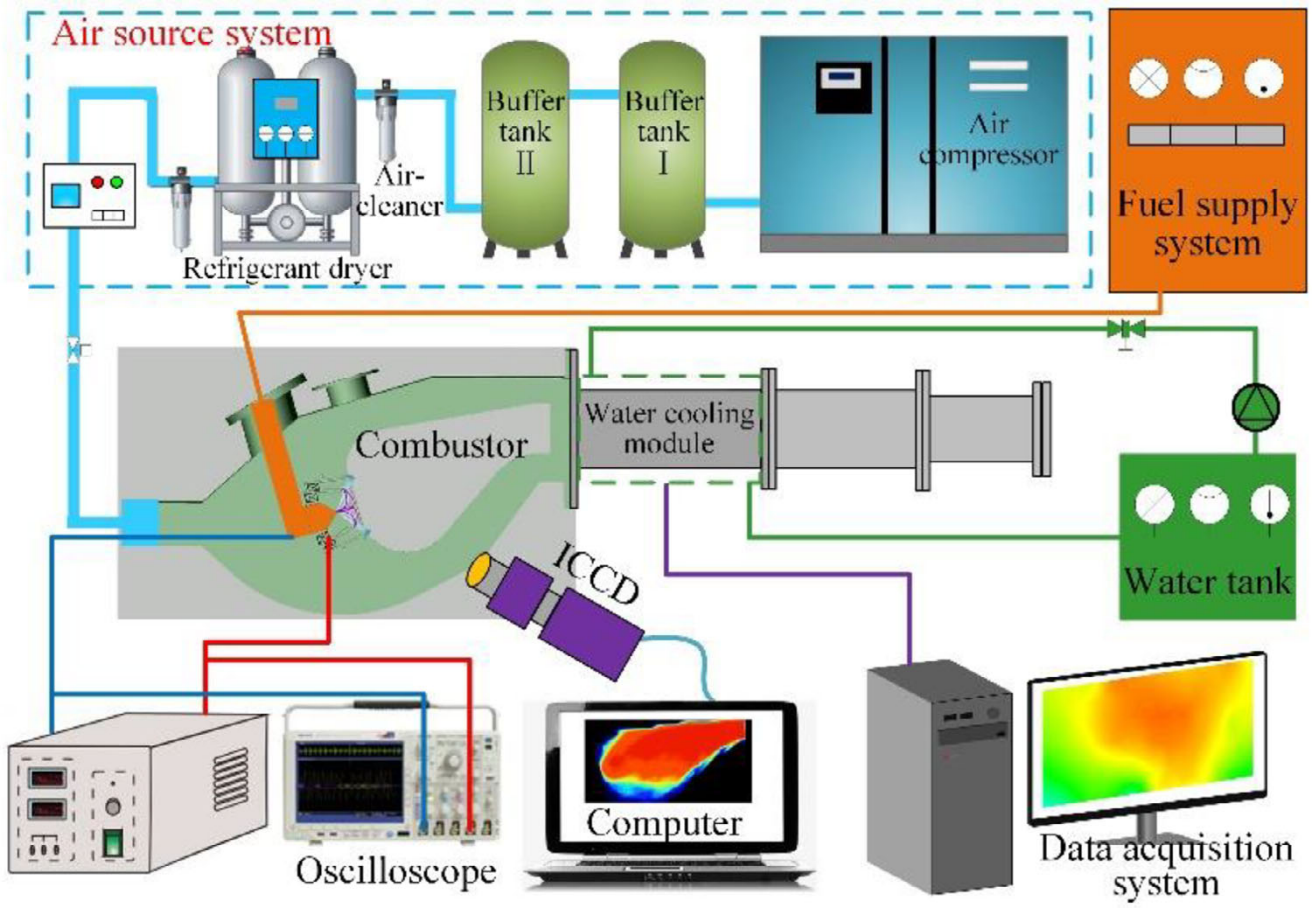
Schematic of experimental system.

**Figure** **10** shows a schematic of the GAP‐assisted combustion experimental system, which mainly consists of a gas source system, fuel supply system, model combustor, measurement and diagnostic system, and control system. The air required during the combustion process is supplied by a screw air compressor (OGFD‐42.8/8B, 250 kW, 42.8 m^3^ min^−1^). The compressed air enters the buffer tank to achieve pressure stability. Finally, it passes through a vortex flowmeter and solenoid valve to achieve precise control (±0.5%) of the inlet flow rate of the combustor. The combustor inlet pressure is measured by a high‐temperature pressure transmitter (PTL701B‐2M, range: 0–2 MPa, accuracy: 0.25% F∙s), and the combustor inlet temperature is measured by a double platinum–rhodium thermocouple (range: 0–1600 °C, accuracy ± 1 °C). The fuel required during the experiment is supplied by a pressure‐based fuel supply system, and is precisely supplied from a storage tank under predetermined operating conditions through a gear flowmeter and pressure‐driven solenoid valve. The control accuracy of the fuel flow rate is 0.001 L min^−1^. The experimental conditions are listed in **Table** **3**. In the table, the excess air coefficient of residual gas is denoted by *α*, representing the ratio of the actual air mass to the theoretical air mass during fuel combustion (α = *m_a_*/(*m_f_* ×*L*_0_); *m*_a_ and *m*_f_ respectively represent the mass flow rates of air and fuel), which reflects the fuel margins in the combustor.

**Table 3:** Experimental conditions.

Parameter (unit)	Value
Fuel type	Aviation kerosene (RP‐3)
Combustor inlet temperature [°C]	27
Combustor inlet pressure [Mpa]	0.25
Combustor inlet velocity [m s^−1^]	10, 15, 20, 25
Excess air coefficient of residual gas	0.8, 1.0, 1.5, 2.0
Gliding arc excitation situation	With GAP/Without GAP

**Figure 11:**
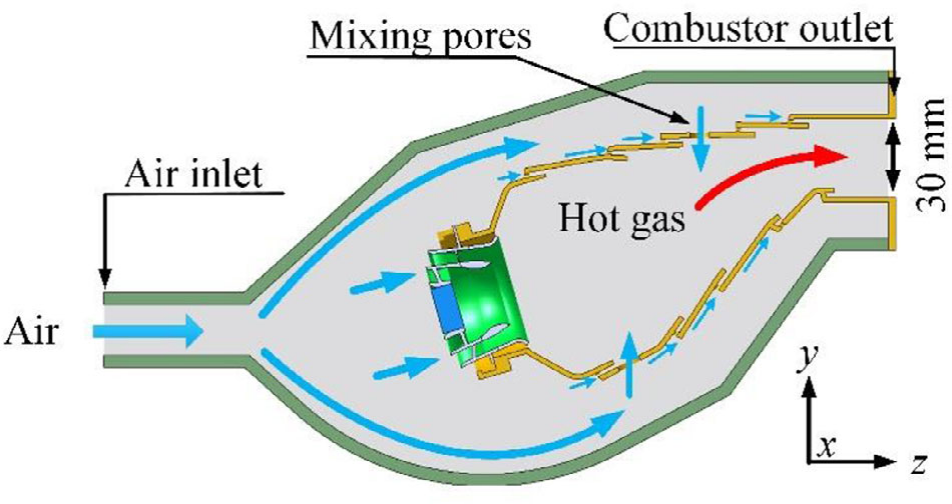
Schematic of combustor structure.

The model combustor is a short annular type designed by the research team. Observation windows on both sides of the combustor are embedded with high‐temperature resistant optical glass to facilitate the acquisition of combustion signals. The outlet size of the combustor is 100 mm × 30 mm and the inlet size of the combustor is 100 mm × 20 mm. A schematic diagram of the combustor structure is shown in **Figure** **11**.

**Figure 12:**
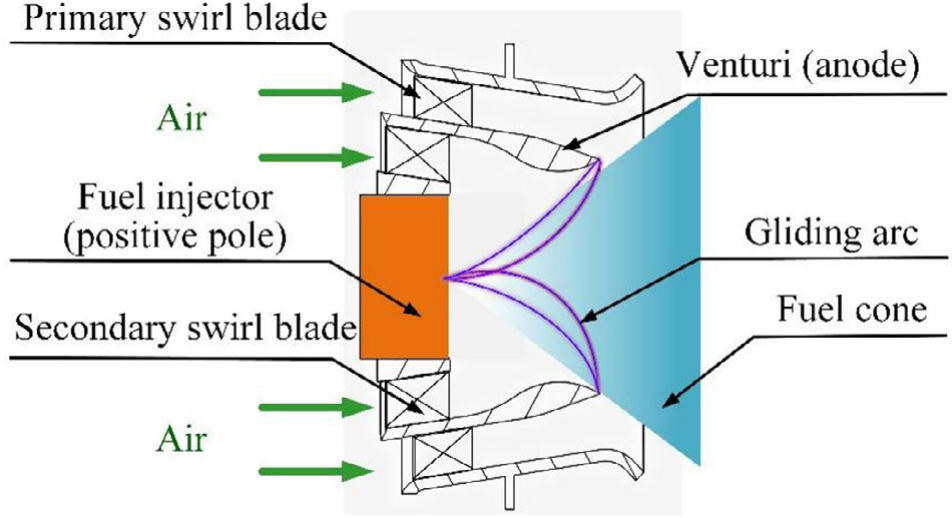
Schematic of the dome structure of the gliding arc plasma combustor.

The gliding arc‐assisted combustion excitation system mainly consists of a GAP combustion dome and driving power supply. The GAP combustion dome is mainly composed of an anode Venturi tube, cathode fuel nozzle, and cyclone substrate;^[^ ^28^, ^29^ ^]^ a structural schematic diagram is shown in **Figure** **12**. The GAP combustion dome is driven by a PG1000‐ZD AC plasma power supply, with an output power range of 300–1000 W and a working frequency of 20 Hz. Once the driving power supply starts working, the anode Venturi tube and cathode fuel nozzle break down under high‐voltage excitation to generate a plasma arc. The plasma arc, driven by a rotating airflow, forms a large and continuous 3D rotating gliding arc discharge at the combustion dome. It should be emphasized that the GAP combustor dome designed in this study is not strictly limited to a dual‐stage axial vortex generator. Its functionality primarily relies on rotational flow‐driven actuation within the combustor. As long as a swirling flow field is present—regardless of the specific combustor architecture—the gliding arc system can be adapted to diverse combustor designs through strategic optimization of electrode geometry and placement.

### Data Collection System

**Figure 13:**
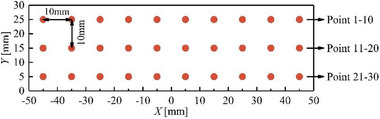
Outlet temperature measuring points in combustor.

The outlet temperature data collection system mainly consists of a National Instruments data acquisition instrument and B‐type thermocouples. The B‐type double platinum–rhodium thermocouples have a temperature measurement range of 250°C–1600°C and can reach 1800°C in a short time; the temperature measurement error is ±1.0°C. Thirty temperature measurement points are arranged at the outlet of the combustor to monitor the temperature in real time. The outlet temperature measurement points are shown in **Figure** **13**. The measuring points are numbered in order from left to right and top to bottom, with point 1 in the upper left corner and point 30 in the lower right corner.

**Figure 14:**
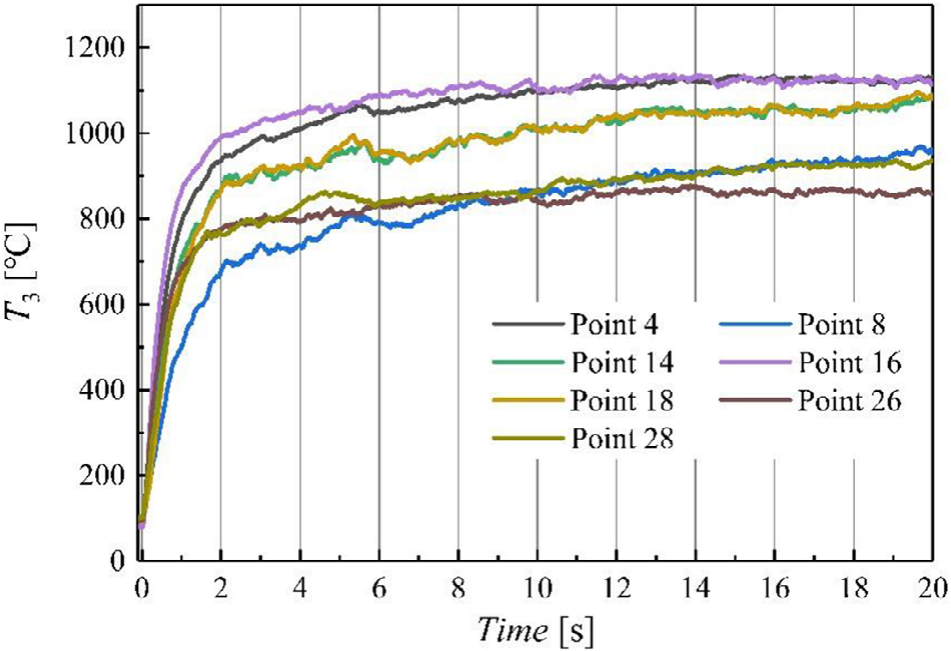
Outlet temperature response time of combustor.

The outlet temperature field of the combustor is nonstationary due to factors such as the inlet flow rate of the combustor, transient excess air coefficient of the combustor, and flame fluctuations. Thus, the outlet temperature of the combustor is collected over an effective time to obtain effective temperature data. **Figure** **14** shows the variation in the outlet temperature of the combustor over time. The response time of the thermocouples is 2 s. The outlet temperature of the combustor tends to stabilize 7 s after ignition. Considering the influence of flame development in the combustor on the outlet temperature, the effective range for collecting the outlet temperature is set to 30–90 s.

**Figure 15:**
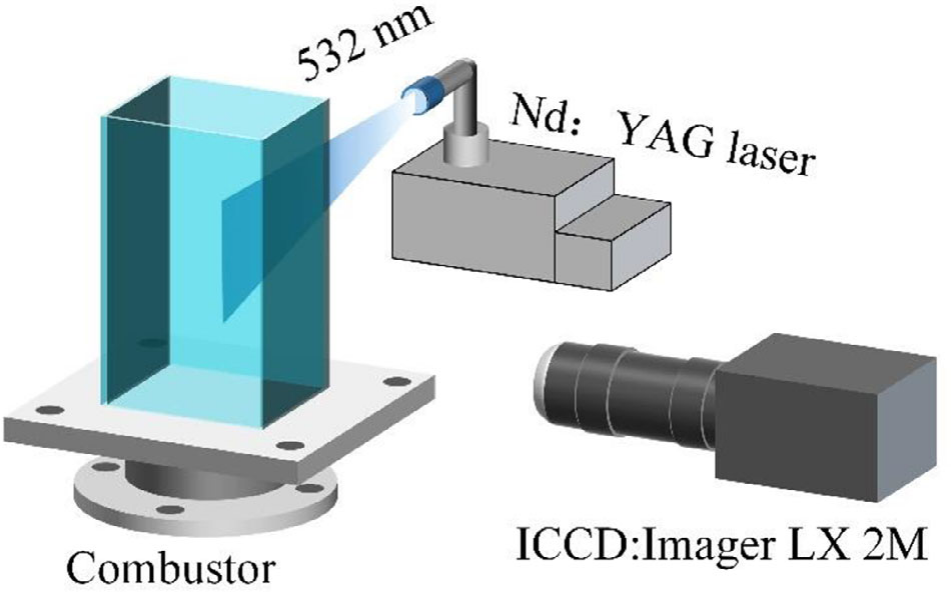
Schematic diagram of PIV measurement system.

PIV technology is used to measure the cold flow field in the swirl combustor. A schematic diagram of the measurement system is shown in **Figure** **15**. In the PIV measurement system, the laser source is an Nd:YAG laser (Litron, UK, wavelength 532 nm, frequency 10 Hz), and the energy of each laser beam is approximately 300 mJ. A CCD camera (Imager LX 2M, resolution 1600×1200, minimum interval between two frames 200 ns) is paired with a macro lens with a focal length of 100 mm aperture F/2.8 to capture the velocity field. The tracer particles are TiO_2_ with a diameter of 0.5 µm. The average velocity field is calculated from 100 instantaneous velocity images. In the cold flow field measurement process, the testing position of PIV is on the plane of *x* = 0 mm, with a testing range of *y* = −50–50 mm and *Z* = 0–100 mm, where the coordinate origin is at the center point of the outlet of the combustor dome.

In combustion reactions, C_2_* groups are generated by the cracking and dissociation of C‐containing alkanes, alkenes, and alkynyl compounds at high temperatures.^[^ ^41^, ^42^ ^]^ C_2_* groups usually represent the heat release of combustion reactions, and the concentration distribution of C_2_* groups is often used to characterize the intensity of combustion reactions and the location of the core reaction zone. Therefore, a high‐speed CCD camera combined with EyeiTS series image intensifiers and 515‐nm band filters forms the flame spontaneous emission acquisition system, which collects C_2_ * group emission signals during the combustion process. To meet the frame size and resolution requirements during the experiment, the acquisition frequency of the high‐speed camera is set to 2500 fps and the gain value of the EyeiTS image intensifier is set to 1500.

### Data Processing Methods

In the combustor of an aeroengine, the uniformity of the outlet temperature profile is usually characterized by OTDF, STDF, and HAR. These parameters are calculated as follows:

(1) $$OTDF=\frac{T_{4\max}-T_{4}}{T_{4}-T_{3}}$$

where *T*_4max _ is the highest gas temperature at the outlet of the combustor, *T*_4_ is the average gas temperature at the outlet of the combustor, and *T*_3_ is the average temperature at the inlet of the combustor. OTDF characterizes the outlet temperature difference of the combustor, with larger values corresponding to worse uniformity of the outlet temperature profile. STDF is calculated as:^[^ ^8^ ^]^

(2) $$STDF=\frac{T_{4l,\max}-T_{4}}{T_{4}-T_{3}}$$

where *T*_4*l*,max _ represents the maximum spanwise average temperature of the combustor outlet. STDF characterizes the difference in spanwise temperature of the combustor outlet; in this article, the spanwise direction represents the *x*‐direction.

(3) $$HAR=\frac{Area(\theta >0.15)}{Area\;measured}$$

where θ = (*T* − *T*_4_)/(*T*_4_ − *T*_3_) is a dimensionless factor that depends on the actual outlet temperature of advanced gas turbine combustors. It is generally believed that θ = 0.15 is the critical value, with θ > 0.15 indicating the hot‐spot area. HAR represents the ratio of the hot‐streak area of the combustor to the overall outlet area of the combustor.

Combustion efficiency characterizes the degree of heat release of fuel in a combustor. The enthalpy increase method calculates combustion efficiency as the ratio of the enthalpy increase of the working fluid at the inlet and outlet of the combustor to the theoretical heat release of the fuel. This method is known to achieve high accuracy. Therefore, in this study, the enthalpy increase method is used to calculate the combustion efficiency of the combustor:^[^ ^6^ ^]^

(4) $$\eta_{c}=\frac{\left(m_{a}+m_{f}\right)c_{p4}T_{4}^{*}-m_{a}c_{p3}T_{3}^{*}-m_{f}c_{pf}T_{f}}{H_{\mu }m_{f}}$$

where *m_a_* and *m_f_* are the mass flow rate of air at the inlet of the combustor (kg s^−1^) and the mass flow rate of fuel (kg s^−1^), respectively. *c*_*p*3_, *c*_*p*4_, and *c_pf_* are the average specific heat at constant pressure of air at the inlet of the combustor (kJ (kg·K) ^−1^), the average specific heat at constant pressure of gas at the outlet of the combustor (kJ (kg·K) ^−1^), and the average specific heat of fuel at the inlet of the combustor (kJ (kg·K) ^−1^). $T_{3}^{*}$ and $T_{4}^{*}$ are the average total temperature of air at the inlet of the combustor and the average total temperature of gas at the outlet of the combustor, respectively. *H*_μ_ is the low heating value of fuel (kJ kg^−1^).

## Conflict of Interest

The authors declare no conflict of interest.

## Data Availability

The data that support the findings of this study are available from the corresponding author upon reasonable request.
